# Neuronal Scaffold Protein ARMS Interacts with Synaptotagmin-4 C2AB through the Ankyrin Repeat Domain with an Unexpected Mode

**DOI:** 10.3390/ijms242316993

**Published:** 2023-11-30

**Authors:** Fa Zhang, Jiasheng Chen, Yahong Li, Jin Ye, Chao Wang

**Affiliations:** 1 Hefei National Research Center for Physical Sciences at the Microscale, MOE Key Laboratory for Membraneless Organelles & Cellular Dynamics, Center for Advanced Interdisciplinary Science and Biomedicine of IHM, Biomedical Sciences and Health Laboratory of Anhui Province, School of Life Sciences, Division of Life Sciences and Medicine, University of Science and Technology of China, Hefei 230027, China; 2 Department of Neurology, The First Affiliated Hospital of USTC, Division of Life Sciences and Medicine, University of Science and Technology of China, Hefei 230027, China

**Keywords:** ARMS, Syt4, BDNF secretion, C2 domain, ankyrin repeats

## Abstract

The ankyrin repeat-rich membrane spanning (ARMS), a transmembrane neuronal scaffold protein, plays a fundamental role in neuronal physiology, including neuronal development, polarity, differentiation, survival and angiogenesis, through interactions with diverse partners. Previous studies have shown that the ARMS negatively regulates brain-derived neurotrophic factor (BDNF) secretion by interacting with Synaptotagmin-4 (Syt4), thereby affecting neurogenesis and the development and function of the nervous system. However, the molecular mechanisms of the ARMS/Syt4 complex assembly remain unclear. Here, we confirmed that the ARMS directly interacts with Syt4 through its N-terminal ankyrin repeats 1–8. Unexpectedly, both the C2A and C2B domains of Syt4 are necessary for binding with the ARMS. We then combined the predicted complex structural models from AlphaFold2 with systematic biochemical analyses using point mutagenesis to underline the molecular basis of ARMS/Syt4 complex formation and to identify two conserved residues, E15 and W72, of the ARMS, as essential residues mediating the assembly of the complex. Furthermore, we showed that ARMS proteins are unable to interact with Syt1 or Syt3, indicating that the interaction between ARMS and Syt4 is specific. Taken together, the findings from this study provide biochemical details on the interaction between the ARMS and Syt4, thereby offering a biochemical basis for the further understanding of the potential mechanisms and functional implications of the ARMS/Syt4 complex formation, especially with regard to the modulation of BDNF secretion and associated neuropathies.

## 1. Introduction

The ankyrin repeat-rich membrane spanning (ARMS), also known as kinase D-interacting substrate of 220 kDa (Kidins220), is a multifunctional scaffolding membrane protein involved in the regulation of a variety of cellular activities, particularly neural differentiation and cytoskeletal remodeling [1,2,3,4]. ARMS is preferentially expressed in the nervous system and binds to Trio, SCG10 and SCLIP to regulate actin and the microtubule cytoskeleton, thus playing a vital role in the coordination of cellular processes such as cell migration, cell polarity and cell cycle regulation [2,5,6]. Evidence has been shown that elevated ARMS protein levels were found in the brains of Alzheimer’s disease (AD) and Huntington’s disease (HD) patients, as well as in related disease models [7,8]. One of the explanations of ARMS-related pathologies is that ARMS is a negative regulator of brain-derived neurotrophic factor (BDNF) secretion in both CNS and PNS neurons [8,9].

ARMS acts as an interaction hub to recruit diverse binders, including neurotrophins and Trk receptors [10], and the depletion of ARMS causes embryonic lethality in mice [11,12,13,14]. BDNF is one of the most widely distributed and extensively studied neurotrophic factors in the mammalian brain. It is critical for neurogenesis and the development and functional maintenance of the nervous system [15,16,17,18,19,20,21], and BDNF levels are found to be reduced in patients or animal models with neurodegenerative diseases such as AD and HD [22,23,24,25]. 

In neurons, endogenous BDNF is mainly stored in dense core vesicles (DCVs), and it is secreted in response to cytoplasmic Ca^2+^ elevation [26,27,28,29,30,31]. Sources that are able to improve this Ca^2+^ concentration to promote BDNF secretion include extracellular Ca^2+^ and the intracellular Ca^2+^ stored in distinct organelles [28,29,30,32]. In addition, BDNF secretion can be triggered by action potentials, nerve growth factor (NGF), neurotrophin-3 (NT-3), neurotrophin-4 (NT-4) and glutamate [8,32,33,34,35,36,37]. It has been reported that the sites of BDNF release are located in the presynaptic membrane, postsynaptic membrane and dendrites [31,36,38,39,40,41,42,43,44,45,46,47,48,49]. However, the molecular mechanism underlying the regulation of BDNF secretion is not yet fully understood.

Studies have found that the levels of ARMS and Synaptotagmin-4 (Syt4) are positively correlated in neurons, and they may directly interact with each other [8]. Syt4 belongs to the synaptotagmin (Syt) family of proteins, which contain seventeen members in humans with distinct intracellular localizations and diverse regulatory functions [50]. Synaptotagmins trigger and regulate the fusion process of vesicles with target membranes, and are thus centrally involved in the precise regulation of neurotransmitter and hormone release processes [50]. In mammals, Syt4 does not bind Ca^2+^, but instead interacts with syntaxin (a component of the SNARE complex) to inhibit SNARE complex-mediated membrane fusion processes [51,52,53,54]. Syt4 is mainly distributed on the membrane of BDNF-carrying DCVs and binds to motor protein Kif1A, which mediates the trafficking of DCVs along microtubules from the soma to the distal dendrites and axons [55,56].

It has been found that Syt4 negatively regulates the secretion of BDNF in neurons, thereby limiting the amplitude of long-term potentiation and synaptic strength, possibly due to the inhibition of the SNARE complex-mediated exocytosis of DCVs [44]. Therefore, it is highly possible that the ARMS regulates BDNF secretion by modulating Syt4 levels, and may thus play a key role in the regulation of nociception and neurodegenerative diseases [8,9]. However, the molecular mechanisms governing ARMS/Syt4 complex formation remain elusive.

Here, we used a combination of molecular biology, biochemistry and structural biology approaches to investigate the assembly mechanisms of the ARMS/Syt4 complex. We first confirmed the direct interaction between ARMS and Syt4 using purified individual proteins. We then mapped the minimal binding regions between ARMS and Syt4 on each protein. Ankyrin repeats 1–8 from the ARMS protein (ARMS R1-8) are responsible for binding with Syt4, and the first ankyrin repeat from ARMS is essential. Meanwhile, neither the C2A nor C2B domains from Syt4 are sufficient for binding with ARMS, giving an unexpected mode that only the C2AB domain could form a complex with ARMS. A series of biochemical analyses using protein variants, together with structure models generated by AlphaFold2, identified key residues essential for the interaction between ARMS and Syt4. Furthermore, we showed that other members of the Syt family (Syt1 and Syt3) could not bind to ARMS. Together, our study provides the biochemical basis of ARMS–Syt4 interaction, and may contribute toward deepening our understanding of the role of the ARMS/Syt4 complex on BDNF secretion and ARMS protein-related neuronal pathologies.

## 2. Results

### 2.1. ARMS Interacts with the Cytoplasmic Region of Syt4 via the Ankyrin Repeat Domain

**Figure 1:**
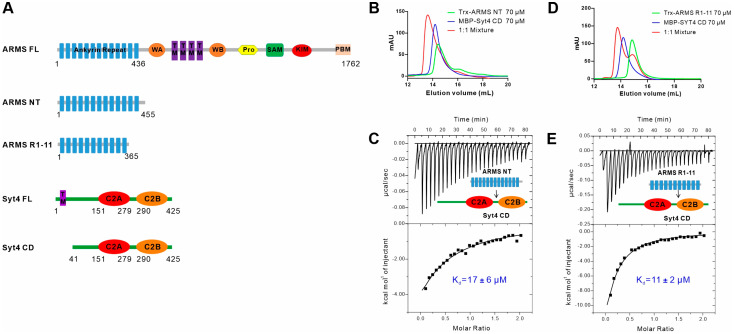
ARMS interacts with the cytoplasmic region of Syt4 via the ankyrin repeat domain. (**A**) A schematic diagram showed the domain organizations of ARMS and Syt4. The full-length ARMS contains ankyrin repeat domain (ARD), a Walker A (WA) motif, a Walker B (WB) motif, four transmembrane fragments (TM), a proline-rich stretch (Pro), a sterile α motif domain (SAM), a kinesin light chain (KLC)-interacting motif (KIM) and a PDZ-binding motif (PBM). The full-length Syt4 contains a transmembrane fragment (TM), a C2A domain and a C2B domain. (**B**,**C**) Analytical gel filtration analysis and isothermal titration calorimetry (ITC) assay showing that ARMS NT (residues 1–455) interacts with Syt4 CD (residues 41–425). (**D**,**E**) Analytical gel filtration analysis and ITC assay showing that ARMS R1-11 (residues 1–365) interacts with Syt4 CD. The K_d_ error is the fitting error obtained using one-site binding kinetics model in Origin 7.0 to fit the ITC data.

Previous studies have found that ARMS interacts with Syt4 through a Co-IP assay, but the interacting regions of the two proteins are unknown. ARMS mainly consists of the N-terminal ankyrin repeat domain (ARD), Walker A and Walker B motifs (WA and WB, respectively) in the juxtamembrane regions flanking the four transmembrane fragments (TM), a proline-rich stretch (Pro), a sterile α motif (SAM) domain, the kinesin light chain (KLC)-interacting motif (KIM) and a PDZ-binding motif located at the C-terminus (Figure 1A). The ARD of ARMS (residues 1–436) is supposed to contain 13 ankyrin repeats and serves as a platform for interaction with a variety of proteins [2,5]. Seeking to characterize the interaction between ARMS and Syt4 in biochemical detail, we conducted an analytical gel filtration chromatography (AGFC) assay to evaluate the binding using a purified ARMS protein N-terminal region (NT, residues 1–455) containing the entire ARD and the full length of the cytoplasmic region of Syt4 (CD, residues 41–425), and showed that the ARMS NT can directly interact with the Syt4 CD, as the 1:1 mixture formed a complex peak (Figure 1B). In addition, we used an isothermal titration calorimetry (ITC) assay to quantify the binding affinity between the ARMS NT and the Syt4 CD. The dissociation constant (K_d_) for the binding was ~17 μM (Figure 1C).

We further truncated the ARMS NT and constructed a fragment of ARMS ankyrin repeats 1–11 (R1-11, residues 1–365) (Figure 1A). Biochemical results showed that ARMS R1-11 could also bind to the Syt4 CD (Figure 1D), and the binding affinity was ~11 μM (Figure 1E), which was similar to the affinity of the ARMS NT to the Syt4 CD, indicating that ARMS R1-11 is sufficient for binding with Syt4. Together these results showed that ARMS interacted with the cytoplasmic region of Syt4 via the ankyrin repeat domain.

### 2.2. C2AB Domain of Syt4 Is the ARMS Binding Region

**Figure 2:**
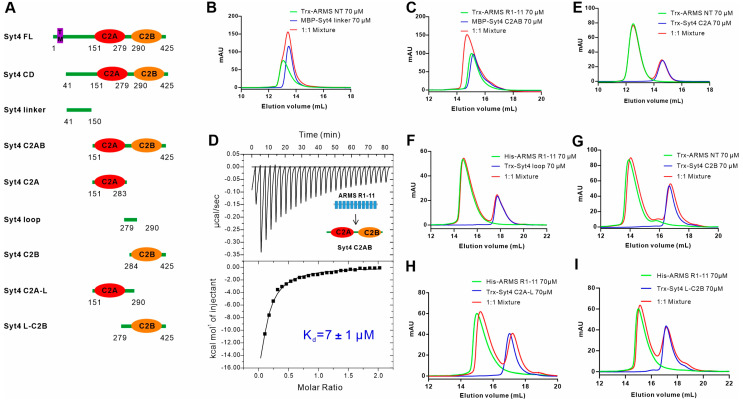
C2AB of Syt4 is the ARMS binding region. (**A**) A schematic diagram of the design of various fragments of the cytoplasmic region of Syt4 (Syt4 CD) based on sequence analysis. (**B**) Analytical gel filtration analysis showing that Syt4 linker (residues 41–150) does not bind to ARMS NT. (**C**,**D**) Analytical gel filtration analysis showing that Syt4 C2AB (residues 151–425) interacts with ARMS R1-11, and the binding affinity of ARMS R1-11 to Syt4 C2AB as measured via ITC. (**E**–**G**) Analytical gel filtration analyses showing that Syt4 C2A (residues 151–283) (**E**), Syt4 loop (residues 279–290) (**F**) and Syt4 C2B (residues 284–425) (**G**) are unable to bind to ARMS protein. (**H**,**I**) Analytical gel filtration analyses showing that Syt4 C2A-L (residues 151–290) (**H**) or Syt4 L-C2B (residues 279–425) (**I**) cannot bind to ARMS R1-11.

Next, we sought out to map the domains of Syt4 that mediate the interaction with ARMS. We designed various fragments of the Syt4 CD based on the sequence analysis of Syt4 structures (Figure 2A), and thereafter assessed the abilities of different regions of Syt4 binding to ARMS using both AGFC and ITC (Figure 2). The results of the AGFC showed that the Syt4 linker (residues 41–150) connecting the transmembrane segment to the C2A domain could not bind to the ARMS NT alone (Figure 2B), while Syt4 C2AB (residues 151–425), containing two C2 domains, bound ARMS R1-11 with a K_d_ of ~7 μM (Figure 2C,D), comparable with the dissociation constant between ARMS R1-11 and the Syt4 CD (~11 μM), indicating that the Syt4 C2AB is responsible for the binding with ARMS. Consequently, we continued to map the minimal binding regions on Syt4. Unexpectedly, we found that any truncations, including the C2A domain (residues 151–283), the loop connecting C2A and C2B (residues 279–290), the C2B domain (residues 284–425), the C2A domain with a loop (C2A-L, residues 151–290), the and C2B domain with a loop (L-C2B, residues 279–425), could not bind with the ARMS NT or R1-11 (Figure 2E–I), suggesting that the ARMS/Syt4 complex assembly required the joint participation of the C2A domain and C2B domain of Syt4. Although the C2 domain can independently serve as a protein–protein interaction module, the coordination or the conformation of the C2A and C2B domains is essential for Syt4 to bind with ARMS. In summary, we found that the binding of Syt4 to ARMS is dependent on its C2AB domain.

### 2.3. ARMS R1-8 Is Required for Binding with Syt4

**Figure 3:**
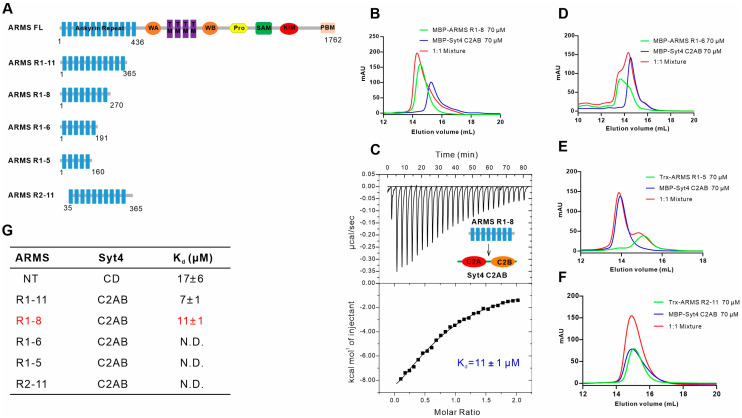
ARMS R1-8 is required for binding with Syt4. (**A**) A schematic diagram of the truncation strategy of ARMS ARD into various fragments with different numbers of ankyrin repeats. (**B**,**C**) Analytical gel filtration analysis showing that ARMS R1-8 (residues 1–270) binds to Syt4 C2AB and the ITC assay for the binding affinity of ARMS R1-8 to Syt4 C2AB. (**D**–**F**) Analytical gel filtration analyses showing that ARMS R1-6 (residues 1-191) (**D**), ARMS R1-5 (residues 1–160) (**E**) or ARMS R2-11 (residues 35–365) (**F**) cannot bind to Syt4 C2AB. (**G**) ITC-based mapping of the minimal Syt4-binding region in ARMS. The minimal Syt4-binding region in ARMS identified is highlighted in red. N.D. denotes that no binding was detected.

Next, we applied a similar strategy to figure out the precise binding region of ARMS for binding with Syt4 by truncating the ARD of ARMS to various fragments containing different numbers of ankyrin repeats (Figure 3A). The interaction between different fragments of the ARMS ARD and Syt4 C2AB was assayed via both AGFC and ITC. We first truncated ARMS R1-11 from the C-terminal and obtained ARMS R1-8 (residues 1–270), ARMS R1-6 (residues 1–191) and ARMS R1-5 (residues 1–160) fragments (Figure 3A). The results of the AGFC and ITC showed that ARMS R1-8 could bind Syt4 C2AB with a binding affinity of ~11 μM, which was similar to ARMS R1-11 and Syt4 C2AB (~7 μM) (Figure 3B,C). The further truncation of the ARD resulted in both ARMS R1-6 and ARMS R1-5 failing to bind to Syt4 C2AB (Figure 3D,E), indicating that ankyrin repeats 7–8 are critical for binding to Syt4, although the underlying basis is unknown. We then truncated ARMS R1-11 from the N-terminal and obtained ARMS R2-11 protein (residues 35–365) by deleting the first ARMS ankyrin repeat (residues 1–34) (Figure 3A). The result of the AGFC clearly showed that ARMS R2-11 does not bind to Syt4 C2AB (Figure 3F), together with the aforementioned data demonstrating the first ankyrin repeat of the ARMS protein is necessary but not sufficient for binding with Syt4. Thus, we concluded that ARMS R1-8 is an effective region required for binding with Syt4 C2AB (Figure 3G). Taken together, we found that the minimal interaction regions for both ARMS and Syt4 are defined as ARMS R1-8 and Syt4 C2AB.

### 2.4. E15 and W72 of ARMS Are Key Residues Mediating the Assembly of the ARMS/Syt4 Complex

**Figure 4:**
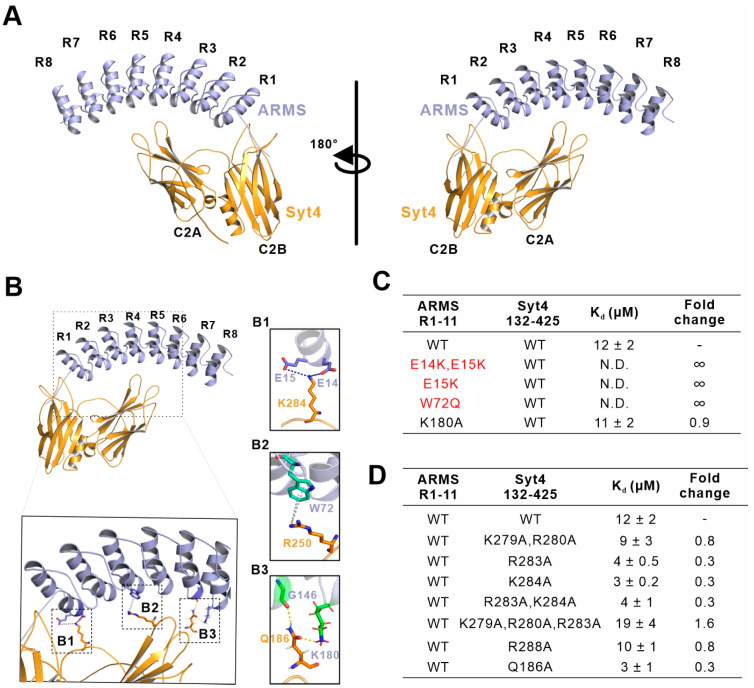
Predicted structural model and interaction analysis of the ARMS/Syt4 complex. (**A**) AlphaFold2_mmseqs2 (version 2.1) prediction of a 1:1 heterodimer composed of rat ARMS (NP_446247.1) residues M1 to D270 and human Syt4 (NP_065834.1) residues E132 to G425 (pLDDT 82, pTM score 0.508 and iptm 0.227). In this drawing, ARMS is shown in light blue and Syt4 is shown in orange. ARMS R1-8 interacts with Syt4 C2AB in an antiparallel manner. (**B**) Ribbon-stick diagram showing that the assemblies of the 3 sites may mediate the binding of ARMS and Syt4 (B1–B3). (**C**) The measured binding affinities between various mutations of ARMS R1-11 and Syt4 132–425 based on ITC assays and comparison against ARMS R1-11 WT in binding with Syt4 132–425. The mutants that break interactions are highlighted in red. N.D. and ∞ indicate that no binding was detected. (**D**) The measured binding affinities between various mutations of Syt4 132–425 and ARMS R1-11 based on ITC assays and comparison against Syt4 132–425 WT in binding with ARMS R1-11.

In order to elucidate the molecular mechanisms governing ARMS and Syt4 complex formation, we first tried to crystallize the complex comprising ARMS protein and Syt4 C2AB. Despite extensive efforts using various ARMS fragments in a complex with Syt4 C2AB or fusion proteins of the ARMS/Syt4 complex, we failed to obtain diffractive crystals. We then turned to using AlphaFold2 to delineate the structural model of the ARMS/Syt4 complex. We derived five structural models of the ARMS/Syt4 complex by inputting the amino acid sequences of ARMS R1-8 and Syt4 132–425 to AlphaFold2_mmseqs2 (version 2.1), which uses models trained on the Protein Structure Database and multiple sequence alignment to infer the structures of proteins and multiprotein complexes. To facilitate our determination of the key residues involved in the formation of the ARMS/Syt4 complex, we selected the highest confidence prediction among five models generated from the AlphaFold2_mmseqs2 analyses (Figure 4A).

The overall predicted structure of the ARMS/Syt4 complex showed that ARMS R1-8 adopted a canonical ankyrin repeat architecture, where each repeat is rotated anticlockwise at an angle to form an interaction inner groove for classical binders (Figure 4A). C2A, C2B and the loop connecting the two C2 domains of Syt4 grabbed the ankyrin repeats like hands to form a complex (Figure 4A). In the interface of the predicted structural model, E14 and E15 from ARMS R1 form electrostatic interactions with K284 on the Syt4 loop (Figure 4(B1)). The sidechain of W72 from ARMS forms a cation–π interaction with R250 from Syt4 (Figure 4(B2)). G146 from ARMS R5 and K180 from ARMS R6 form hydrogen bonds with Q186 from Syt4 C2A (Figure 4(B1)). Furthermore, we verified whether these amino acids could affect the binding of ARMS to Syt4 by point-mutating the possible binding sites and testing the effect of these mutations on the interaction using ITC. Notably, we use ARMS R1-11 and Syt4 132–425 (more stable than Syt4 C2AB) as references in the following ITC experiments (Figure 4C,D and Figure S1A).

The ITC data showed that the ARMS bearing the E14K and E15K variants resulted in binding to Syt4 being abolished, and a single variant of E15K of the ARMS protein disrupted the binding, suggesting the vital role of E15 from ARMS protein (Figure 4C and Figure S1). However, the Syt4 K284A variant did not break or weaken the binding (Figure 4D and Figure S2), and mutating the nearby R279, R280, R283, and R288 to Ala did not weaken the binding of the ARMS protein and Syt4 (Figure 4D and Figure S2). The reason may be that these positively charged residues including R279, R280, R283, and R288 could compensate for the effects produced by the single or combinations of the mutations. The ARMS W72Q variant does not bind Syt4, indicating that W72 is also a key residue for binding to Syt4 (Figure 4C and Figure S1). In addition, neither the ARMS K180A nor Syt4 Q186A variants could break or weaken the binding between ARMS and Syt4, indicating that K180 from ARMS and Q186 from Syt4 are not involved in the interaction (Figure 4C,D and Figures S1 and S2). Our biochemical data, together with the predicted structural model, showed that E15 and W72 of ARMS are key residues in the assembly of the ARMS/Syt4 complex.

### 2.5. Multiple Sequence Alignment Reveals Highly Conserved Residues

**Figure 5:**
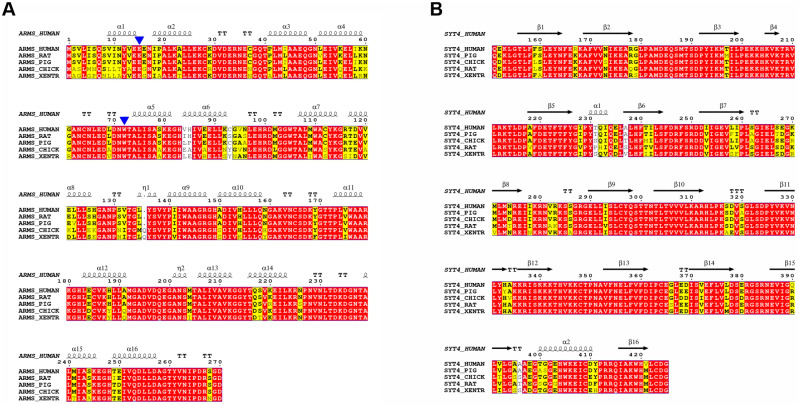
Multiple sequence alignments of ARMS and Syt4. Multi-sequence alignment of ARMS R1-8 (**A**) and Syt4 C2AB (**B**) across different species. The conserved residues are colored yellow, and identical residues are colored red. E15 and W72 are labeled with blue triangles.

Amino acid sequence alignment analyses showed that ARMS R1-8 and Syt4 CAB are highly conserved across a wide range of different species, indicating that ARMS R1-8/Syt4 CAB interactions have retained a selective advantage during evolution (Figure 5A,B). Notably, E15 and W72, which are essential binding sites for Syt4, are conserved across ARMS from different species (Figure 5A). Collectively, these results provide a mechanistic explanation for the binding of ARMS ARD to Syt4 CAB.

### 2.6. ARMS Does Not Bind to Syt1 or Syt3

**Figure 6:**
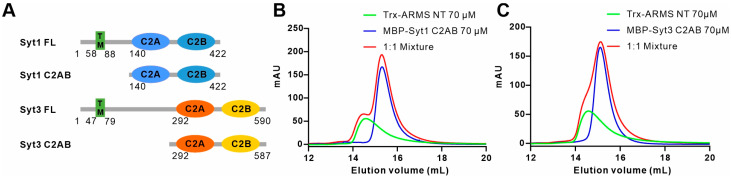
ARMS does not bind to Syt1 or Syt3. (**A**) A schematic diagram showing the domain organization and the truncation strategy of Syt1 and Syt3. (**B**,**C**) Analytical gel filtration analyses showing that Syt1 (**B**) and Syt3 (**C**) cannot bind to ARMS NT.

While Syt4 does not directly bind Ca^2+^, both Syt1 and Syt3 are intra-neuronal calcium sensors involved in membrane fusion processes [50,57]. Syt1 can bind Ca^2+^ through two C2 domains and positively regulates the exocytosis of synaptic vesicles (SVs) and DCVs catalyzed via the SNARE complex [50,58,59]. On the other hand, Syt3 is distributed in both presynaptic and postsynaptic membranes, and its function varies depending on its location. Presynaptic Syt3 drives vesicle replenishment and short-term synaptic plasticity and helps to replenish the supply of neurotransmitters, playing a key role in maintaining high-frequency synaptic transmission [57]. Postsynaptic Syt3 triggers the calcium-mediated internalization of AMPA receptors, leading to impaired synaptic transmission and spatial memory forgetting in mice [60]. To explore whether ARMS can bind other members of the Syt family, such as Syt1 and Syt3, we purified the Syt1 and Syt3 C2AB domain proteins, respectively (Figure 6A). The results of the AGFC showed that both Syt1 C2AB (residues 140–422) and Syt3 C2AB (residues 292–587) failed to bind to the ARMS NT (Figure 6B,C), demonstrating that the interaction between ARMS and Syt4 is not universal for all members of the Syt family.

## 3. Discussion

In this study, we used biochemical methods, including AGFC and ITC, to identify the optimal regions where ARMS interacts with Syt4, and mapped out ankyrin repeats 1–8 of ARMS and the C2AB domain of Syt4 as the binding sites. In addition, the results of multiple sequence comparisons showed that ankyrin repeats 1–8 of the ARMS protein and the C2AB domain of Syt4 are highly conserved during evolution. We then performed a structural prediction of the ARMS/Syt4 complex and made point mutations that may be involved in the interaction based on the predicted structural model of the complex. A biochemical analysis confirmed that E15 and W72 of ARMS are essential residues mediating binding to Syt4, and they are highly conserved in different species. However, residues of Syt4 C2AB that pair with the binding sites of ARMS and the binding sites in ARMS R7-8 were not confirmed from the structural model. Therefore, the structure of the ARMS/Syt4 complex predicted using AlphaFold2 cannot be fully accurate at characterizing the interfaces and the detailed binding mode of the ARMS/Syt4 complex. There may be residues on ARMS R1-8 and Syt4 C2AB that may be involved in the binding that have not yet been discovered. It may be necessary to obtain the real structure of the ARMS/Syt4 complex in order to address the mechanistic gaps, especially with regard to pathologies.

In neurons, ARMS locates on the plasma membranes of the whole soma, dendrites and axons, and the positions of DCV exocytosis and BDNF secretion are located on the plasma membranes of dendritic spines and synaptosomes [4,31,32,33]. Therefore, we hypothesize that, in dendritic spines and synaptosomes, ARMS recruits Syt4 to the vicinity of the plasma membranes and blocks the assembly of the SNARE complex, thereby inhibiting the SNARE complex-mediated exocytosis of DCVs and reducing the secretion of BDNF. However, proof of the specific co-localization of ARMS with Syt4 in neurons beneath the plasma membranes of dendritic spines and synaptosomes, as well as the disruption of their interaction to facilitate BDNF secretion, is needed to confirm this hypothesis. Both the E15K and W72Q variants of ARMS break the binding of ARMS to Syt4, so these mutants can be applied in subsequent functional studies to explore the localization of ARMS and Syt4 in neurons and verify the role of the ARMS/Syt4 complex in the regulation of BDNF secretion.

Although the tertiary structure of the C2A domain and the C2B domain of Syt family members is similar, Syt1 and Syt3, both belonging to the Syt family, cannot bind to ARMS like Syt4 can. Moreover, the differences in the relative orientation of the C2A domain and the C2B domain may also be responsible for the distinction in biochemical characteristics and functions among Syt1, Syt3 and Syt4. The binding of ARMS to Syt4 may be specific, but further confirmation of this idea requires biochemical testing of the interactions between other members of the Syt family and ARMS. In conclusion, our study establishes a biochemical basis for understanding the formation of the ARMS/Syt4 complex, which is important for gaining insight into the mechanisms of the regulation of BDNF secretion and neurodegenerative diseases.

## 4. Materials and Methods

### 4.1. Constructs, Protein Expression, and Purification

The gene encoding of a full-length ARMS protein (UniProt: Q9EQG6) was PCR-amplified using rat cDNA as the template. A full-length Syt1 gene (UniProt: P21579), full-length Syt3 gene (UniProt: Q9BQG1) and full-length Syt4 gene (UniProt: Q9H2B2) were PCR-amplified with a human cDNA library as the template. Various mutations or shorter fragments of the ARMS protein, Syt1, Syt3 and Syt4 were generated using standard PCR-based methods and confirmed via DNA sequencing. All of the protein constructs were cloned into pET32a, pET.M.3C or pETDuet-1 for protein expression and confirmed via DNA sequencing. The pET32a vector is a home-modified version with a thioredoxin (Trx)-His_6_ tag or a maltose binding protein (MBP)-His_6_ tag. All the proteins were expressed in *Escherichia coli* BL21 (DE3) cells. The proteins with an N-terminal Trx-His_6_-tag, MBP-His_6_-tag or His_6_-tag were purified using an Ni-NTA agarose affinity column followed by size-exclusion chromatography (Superdex 200 column, GE Healthcare, Chicago, IL, USA) in a buffer containing 50 mM of Tris, 100 mM of NaCl, 1 mM of EDTA and 1 mM of DTT at pH 7.8.

### 4.2. Analytical Gel Filtration Chromatography Assay

Analytical gel filtration chromatography (AGFC) was carried out on an AKTA Pure system (GE Healthcare). The proteins were loaded onto a Superose 12 column (GE Healthcare) or a Superdex 200 increase column equilibrated with a buffer containing 50 mM of Tris, 100 mM of NaCl, 1 mM of EDTA and 1 mM of DTT at pH 7.8. All graphs were drawn using GraphPad Prism 8 (GraphPad Software, La Jolla, CA, USA).

### 4.3. Isothermal Titration Calorimetry Assay

Isothermal titration calorimetry (ITC) measurements were carried out on a VP-ITC MicroCal calorimeter (Malvern Panalytical Ltd, Malvern, UK) at 25 °C. All proteins were dissolved in the buffer containing 50 mM of Tris, 100 mM of NaCl, 1 mM of EDTA and 1 mM of DTT at pH 7.8. The ARMS proteins (200 μM) were loaded into a syringe, and the Syt4 proteins (20 μM) were loaded in the cell. Each titration point was obtained by injecting a 10 μL aliquot of the syringe protein into the cell at a time interval of 180 s to ensure that the titration peak returned to the baseline. The titration data were analyzed using the Origin 7.0 (Microcal, Malvern Panalytical Ltd., Malvern, UK) program and fitted using a one-site binding model to determine the binding affinities.

### 4.4. Structure Prediction

Predictions of complex structures were made using the Google Colab AlphafoldNotebook (AlphaFold2_mmseqs2) with a Colab Pro+ subscription “https://colab.research.google.com/github/sokrypton/ColabFold/blob/main/AlphaFold2.ipynb” (accessed on 20 February 2023). The algorithm was tasked to model a 1:1 heterodimer, comprising residues M1 to D270 of rat ARMS proteins (UniProt: Q9EQG6) and residues E132 to G425 of human Syt4 (UniProt: Q9H2B2). Default parameters were employed, with the amber relaxation step activated, subsequently generating the outcomes of five predictive models. For our study, we selected the suitable model that represented the highest confidence prediction among the five models. The optimal model was downloaded in a PDB format and prepared for display using either PyMOL (https://pymol.org/2/) (accessed on 20 February 2023) or UCSF Chimera (https://www.cgl.ucsf.edu/chimera/) (accessed on 20 February 2023).

### 4.5. Sequence Alignment

The sequence of the human ARMS proteins and human Syt4 were obtained from the UniProt database, and these two sequence were submitted to the NCBI-BLAST to search for homologs. A total of 5 homologous sequences were selected and aligned using multiple sequence alignment (http://multalin.toulouse.inra.fr/multalin/) (accessed on 20 February 2023) with default parameters. The ESpript 3.0 software was used to generate the color-coded version of the multiple sequence alignment (http://espript.ibcp.fr/ESPript/cgi-bin/ESPript.cgit) (accessed on 20 February 2023).

## Data Availability

Data is contained within the article and supplementary material.

## Supplementary Materials

The following supporting information can be downloaded at https://www.mdpi.com/article/10.3390/ijms242316993/s1.

## References

[1] Bracale A., Cesca F., Neubrand V.E., Newsome T.P., Way M., Schiavo G. (2007). Kidins220/ARMS Is Transported by a Kinesin-1-Based Mechanism Likely to Be Involved in Neuronal Differentiation. Mol. Biol. Cell.

[2] Higuero A.M., Sánchez-Ruiloba L., Doglio L.E., Portillo F., Abad-Rodríguez J., Dotti C.G., Iglesias T. (2010). Kidins220/ARMS Modulates the Activity of Microtubule-Regulating Proteins and Controls Neuronal Polarity and Development. J. Biol. Chem..

[3] Neubrand V.E., Thomas C., Schmidt S., Debant A., Schiavo G. (2010). Kidins220/ARMS Regulates Rac1-Dependent Neurite Outgrowth by Direct Interaction with the RhoGEF Trio. J. Cell Sci..

[4] Scholz-Starke J., Cesca F. (2016). Stepping Out of the Shade: Control of Neuronal Activity by the Scaffold Protein Kidins220/ARMS. Front. Cell. Neurosci..

[5] Cai S., Cai J., Jiang W.G., Ye L. (2017). Kidins220 and Tumour Development: Insights into a Complexity of Cross-Talk among Signalling Pathways (Review). Int. J. Mol. Med..

[6] Fife C.M., McCarroll J.A., Kavallaris M. (2014). Movers and Shakers: Cell Cytoskeleton in Cancer Metastasis. Br. J. Pharmacol..

[7] López-Menéndez C., Gamir-Morralla A., Jurado-Arjona J., Higuero A.M., Campanero M.R., Ferrer I., Hernández F., Ávila J., Díaz-Guerra M., Iglesias T. (2013). Kidins220 Accumulates with Tau in Human Alzheimer’s Disease and Related Models: Modulation of Its Calpain-Processing by GSK3β/PP1 Imbalance. Hum. Mol. Genet..

[8] López-Benito S., Sánchez-Sánchez J., Brito V., Calvo L., Lisa S., Torres-Valle M., Palko M.E., Vicente-García C., Fernández-Fernández S., Bolaños J.P. (2018). Regulation of BDNF Release by ARMS/Kidins220 through Modulation of Synaptotagmin-IV Levels. J. Neurosci..

[9] Sánchez-Sánchez J., Vicente-García C., Cañada-García D., Martín-Zanca D., Arévalo J.C. (2023). ARMS/Kidins220 Regulates Nociception by Controlling Brain-Derived Neurotrophic Factor Secretion. Pain.

[10] Neubrand V.E., Cesca F., Benfenati F., Schiavo G. (2012). Kidins220/ARMS as a Functional Mediator of Multiple Receptor Signalling Pathways. J. Cell Sci..

[11] Cesca F., Yabe A., Spencer-Dene B., Scholz-Starke J., Medrihan L., Maden C.H., Gerhardt H., Orriss I.R., Baldelli P., Al-Qatari M. (2012). Kidins220/ARMS Mediates the Integration of the Neurotrophin and VEGF Pathways in the Vascular and Nervous Systems. Cell Death Differ..

[12] Mero I.-L., Mørk H.H., Sheng Y., Blomhoff A., Opheim G.L., Erichsen A., Vigeland M.D., Selmer K.K. (2017). Homozygous KIDINS220 Loss-of-Function Variants in Fetuses with Cerebral Ventriculomegaly and Limb Contractures. Hum. Mol. Genet..

[13] El-Dessouky S.H., Issa M.Y., Aboulghar M.M., Gaafar H.M., Elarab A.E., Ateya M.I., Omar H.H., Beetz C., Zaki M.S. (2020). Prenatal Delineation of a Distinct Lethal Fetal Syndrome Caused by a Homozygous Truncating KIDINS220 Variant. Am. J. Med. Genet. A.

[14] Wu S.H., Arévalo J.C., Sarti F., Tessarollo L., Gan W.-B., Chao M.V. (2009). ARMS/Kidins220 Regulates Dendritic Branching and Spine Stability in Vivo. Dev. Neurobiol..

[15] Colucci-D’Amato L., Speranza L., Volpicelli F. (2020). Neurotrophic Factor BDNF, Physiological Functions and Therapeutic Potential in Depression, Neurodegeneration and Brain Cancer. IJMS.

[16] Park H., Poo M. (2013). Neurotrophin Regulation of Neural Circuit Development and Function. Nat. Rev. Neurosci..

[17] Bartkowska K., Paquin A., Gauthier A.S., Kaplan D.R., Miller F.D. (2007). Trk Signaling Regulates Neural Precursor Cell Proliferation and Differentiation during Cortical Development. Development.

[18] Snapyan M., Lemasson M., Brill M.S., Blais M., Massouh M., Ninkovic J., Gravel C., Berthod F., Götz M., Barker P.A. (2009). Vasculature Guides Migrating Neuronal Precursors in the Adult Mammalian Forebrain via Brain-Derived Neurotrophic Factor Signaling. J. Neurosci..

[19] Scharfman H., Goodman J., Macleod A., Phani S., Antonelli C., Croll S. (2005). Increased Neurogenesis and the Ectopic Granule Cells after Intrahippocampal BDNF Infusion in Adult Rats. Exp. Neurol..

[20] Lee J., Duan W., Mattson M.P. (2002). Evidence That Brain-Derived Neurotrophic Factor Is Required for Basal Neurogenesis and Mediates, in Part, the Enhancement of Neurogenesis by Dietary Restriction in the Hippocampus of Adult Mice. J. Neurochem..

[21] Rossi C., Angelucci A., Costantin L., Braschi C., Mazzantini M., Babbini F., Fabbri M.E., Tessarollo L., Maffei L., Berardi N. (2006). Brain-Derived Neurotrophic Factor (BDNF) Is Required for the Enhancement of Hippocampal Neurogenesis Following Environmental Enrichment. Eur. J. Neurosci..

[22] Hock C., Heese K., Hulette C., Rosenberg C., Otten U. (2000). Region-Specific Neurotrophin Imbalances in Alzheimer Disease: Decreased Levels of Brain-Derived Neurotrophic Factor and Increased Levels of Nerve Growth Factor in Hippocampus and Cortical Areas. Arch. Neurol..

[23] Ferrer I., Goutan E., Marín C., Rey M.J., Ribalta T. (2000). Brain-Derived Neurotrophic Factor in Huntington Disease. Brain Res..

[24] Zuccato C., Marullo M., Conforti P., MacDonald M.E., Tartari M., Cattaneo E. (2008). Systematic Assessment of BDNF and Its Receptor Levels in Human Cortices Affected by Huntington’s Disease. Brain Pathol..

[25] Narisawa-Saito M., Wakabayashi K., Tsuji S., Takahashi H., Nawa H. (1996). Regional Specificity of Alterations in NGF, BDNF and NT-3 Levels in Alzheimer’s Disease. NeuroReport.

[26] Dieni S., Matsumoto T., Dekkers M., Rauskolb S., Ionescu M.S., Deogracias R., Gundelfinger E.D., Kojima M., Nestel S., Frotscher M. (2012). BDNF and Its Pro-Peptide Are Stored in Presynaptic Dense Core Vesicles in Brain Neurons. J. Cell Biol..

[27] Berg E.A., Johnson R.J., Leeman S.E., Boyd N., Kimerer L., Fine R.E. (2000). Isolation and Characterization of Substance P-Containing Dense Core Vesicles from Rabbit Optic Nerve and Termini. J. Neurosci. Res..

[28] Canossa M., Gärtner A., Campana G., Inagaki N., Thoenen H. (2001). Regulated Secretion of Neurotrophins by Metabotropic Glutamate Group I (mGluRI) and Trk Receptor Activation Is Mediated via Phospholipase C Signalling Pathways. EMBO J.

[29] Goodman L.J., Valverde J., Lim F., Geschwind M.D., Federoff H.J., Geller A.I., Hefti F. (1996). Regulated Release and Polarized Localization of Brain-Derived Neurotrophic Factor in Hippocampal Neurons. Mol. Cell Neurosci..

[30] Griesbeck O., Canossa M., Campana G., Gärtner A., Hoener M.C., Nawa H., Kolbeck R., Thoenen H. (1999). Are There Differences between the Secretion Characteristics of NGF and BDNF? Implications for the Modulatory Role of Neurotrophins in Activity-Dependent Neuronal Plasticity. Microsc Res Tech.

[31] Matsuda N., Lu H., Fukata Y., Noritake J., Gao H., Mukherjee S., Nemoto T., Fukata M., Poo M. (2009). Differential Activity-Dependent Secretion of Brain-Derived Neurotrophic Factor from Axon and Dendrite. J. Neurosci..

[32] Arévalo J.C., Deogracias R. (2023). Mechanisms Controlling the Expression and Secretion of BDNF. Biomolecules.

[33] Canossa M., Griesbeck O., Berninger B., Campana G., Kolbeck R., Thoenen H. (1997). Neurotrophin Release by Neurotrophins: Implications for Activity-Dependent Neuronal Plasticity. Proc. Natl. Acad. Sci. USA.

[34] Krüttgen A., Möller J.C., Heymach J.V., Shooter E.M. (1998). Neurotrophins Induce Release of Neurotrophins by the Regulated Secretory Pathway. Proc. Natl. Acad. Sci. USA.

[35] Leßmann V. (1998). Neurotrophin-Dependent Modulation of Glutamatergic Synaptic Transmission in the Mammalian CNS. Gen. Pharmacol. Vasc. Syst..

[36] Kuczewski N., Porcher C., Ferrand N., Fiorentino H., Pellegrino C., Kolarow R., Lessmann V., Medina I., Gaiarsa J.-L. (2008). Backpropagating Action Potentials Trigger Dendritic Release of BDNF during Spontaneous Network Activity. J. Neurosci..

[37] Edelmann E., Leßmann V., Brigadski T. (2014). Pre- and Postsynaptic Twists in BDNF Secretion and Action in Synaptic Plasticity. Neuropharmacol..

[38] Hartmann M., Heumann R., Lessmann V. (2001). Synaptic Secretion of BDNF after High-Frequency Stimulation of Glutamatergic Synapses. EMBO J.

[39] Brigadski T., Hartmann M., Lessmann V. (2005). Differential Vesicular Targeting and Time Course of Synaptic Secretion of the Mammalian Neurotrophins. J. Neurosci..

[40] Kojima M., Takei N., Numakawa T., Ishikawa Y., Suzuki S., Matsumoto T., Katoh-Semba R., Nawa H., Hatanaka H. (2001). Biological Characterization and Optical Imaging of Brain-Derived Neurotrophic Factor-Green Fluorescent Protein Suggest an Activity-Dependent Local Release of Brain-Derived Neurotrophic Factor in Neurites of Cultured Hippocampal Neurons. J. Neurosci. Res..

[41] Kolarow R., Brigadski T., Lessmann V. (2007). Postsynaptic Secretion of BDNF and NT-3 from Hippocampal Neurons Depends on Calcium–Calmodulin Kinase II Signaling and Proceeds via Delayed Fusion Pore Opening. J. Neurosci..

[42] Lochner J.E., Spangler E., Chavarha M., Jacobs C., McAllister K., Schuttner L.C., Scalettar B.A. (2008). Efficient Copackaging and Cotransport Yields Postsynaptic Colocalization of Neuromodulators Associated with Synaptic Plasticity. Dev. Neurobiol..

[43] Rind H.B., Butowt R., Bartheld C.S. (2005). von Synaptic Targeting of Retrogradely Transported Trophic Factors in Motoneurons: Comparison of Glial Cell Line-Derived Neurotrophic Factor, Brain-Derived Neurotrophic Factor, and Cardiotrophin-1 with Tetanus Toxin. J. Neurosci..

[44] Dean C., Liu H., Mark Dunning F., Chang P.Y., Jackson M.B., Chapman E.R. (2009). Synaptotagmin-IV Modulates Synaptic Function and Long-Term Potentiation by Regulating BDNF Release. Nat Neurosci.

[45] Haubensak W., Narz F., Heumann R., Leβmann V. (1998). BDNF-GFP Containing Secretory Granules Are Localized in the Vicinity of Synaptic Junctions of Cultured Cortical Neurons. J. Cell Sci..

[46] Kohara K., Kitamura A., Morishima M., Tsumoto T. (2001). Activity-Dependent Transfer of Brain-Derived Neurotrophic Factor to Postsynaptic Neurons. Science.

[47] Sadakata T., Shinoda Y., Oka M., Sekine Y., Sato Y., Saruta C., Miwa H., Tanaka M., Itohara S., Furuichi T. (2012). Reduced Axonal Localization of a Caps2 Splice Variant Impairs Axonal Release of BDNF and Causes Autistic-like Behavior in Mice. Proc. Natl. Acad. Sci. USA.

[48] Scalettar B.A., Jacobs C., Fulwiler A., Prahl L., Simon A., Hilken L., Lochner J.E. (2012). Hindered Submicron Mobility and Long-Term Storage of Presynaptic Dense-Core Granules Revealed by Single-Particle Tracking. Dev. Neurobiol..

[49] Shinoda Y., Sadakata T., Nakao K., Katoh-Semba R., Kinameri E., Furuya A., Yanagawa Y., Hirase H., Furuichi T. (2011). Calcium-Dependent Activator Protein for Secretion 2 (CAPS2) Promotes BDNF Secretion and Is Critical for the Development of GABAergic Interneuron Network. Proc. Natl. Acad. Sci. USA.

[50] Wolfes A.C., Dean C. (2020). The Diversity of Synaptotagmin Isoforms. Curr. Opin. Neurobiol..

[51] Ahras M., Otto G.P., Tooze S.A. (2006). Synaptotagmin IV Is Necessary for the Maturation of Secretory Granules in PC12 Cells. J. Cell Biol..

[52] Dai H., Shin O.-H., Machius M., Tomchick D.R., Südhof T.C., Rizo J. (2004). Structural Basis for the Evolutionary Inactivation of Ca2+ Binding to Synaptotagmin 4. Nat. Struct. Mol. Biol..

[53] Thomas D.M., Ferguson G.D., Herschman H.R., Elferink L.A. (1999). Functional and Biochemical Analysis of the C2 Domains of Synaptotagmin IV. MBoC.

[54] Wang Z., Chapman E.R. (2010). Rat and Drosophila Synaptotagmin 4 Have Opposite Effects during SNARE-Catalyzed Membrane Fusion. J. Biol. Chem..

[55] Bharat V., Siebrecht M., Burk K., Ahmed S., Reissner C., Kohansal-Nodehi M., Steubler V., Zweckstetter M., Ting J.T., Dean C. (2017). Capture of Dense Core Vesicles at Synapses by JNK-Dependent Phosphorylation of Synaptotagmin-4. Cell Rep..

[56] McVicker D.P., Awe A.M., Richters K.E., Wilson R.L., Cowdrey D.A., Hu X., Chapman E.R., Dent E.W. (2016). Transport of a Kinesin-Cargo Pair along Microtubules into Dendritic Spines Undergoing Synaptic Plasticity. Nat. Commun..

[57] Weingarten D.J., Shrestha A., Juda-Nelson K., Kissiwaa S.A., Spruston E., Jackman S.L. (2022). Fast Resupply of Synaptic Vesicles Requires Synaptotagmin-3. Nature.

[58] Van Westen R., Poppinga J., Díez Arazola R., Toonen R.F., Verhage M. (2021). Neuromodulator Release in Neurons Requires Two Functionally Redundant Calcium Sensors. Proc. Natl. Acad. Sci. USA.

[59] Zhou Q., Zhou P., Wang A.L., Wu D., Zhao M., Südhof T.C., Brunger A.T. (2017). The Primed SNARE–Complexin–Synaptotagmin Complex for Neuronal Exocytosis. Nature.

[60] Awasthi A., Ramachandran B., Ahmed S., Benito E., Shinoda Y., Nitzan N., Heukamp A., Rannio S., Martens H., Barth J. (2019). Synaptotagmin-3 Drives AMPA Receptor Endocytosis, Depression of Synapse Strength, and Forgetting. Science.

