# Supplementary material for: Neuronal Scaffold Protein ARMS Interacts with Synaptotagmin-4 C2AB through the Ankyrin Repeat Domain with an Unexpected Mode
Source: Int J Mol Sci. 2023 Nov 30;24(23):16993. doi: 10.3390/ijms242316993 (PMC10707181; doi:10.3390/ijms242316993)
Supplement: Supplementary file 1 [file ijms-24-16993-s001.zip › ijms-2609655-supplementary.pdf]

Supplementary Materials

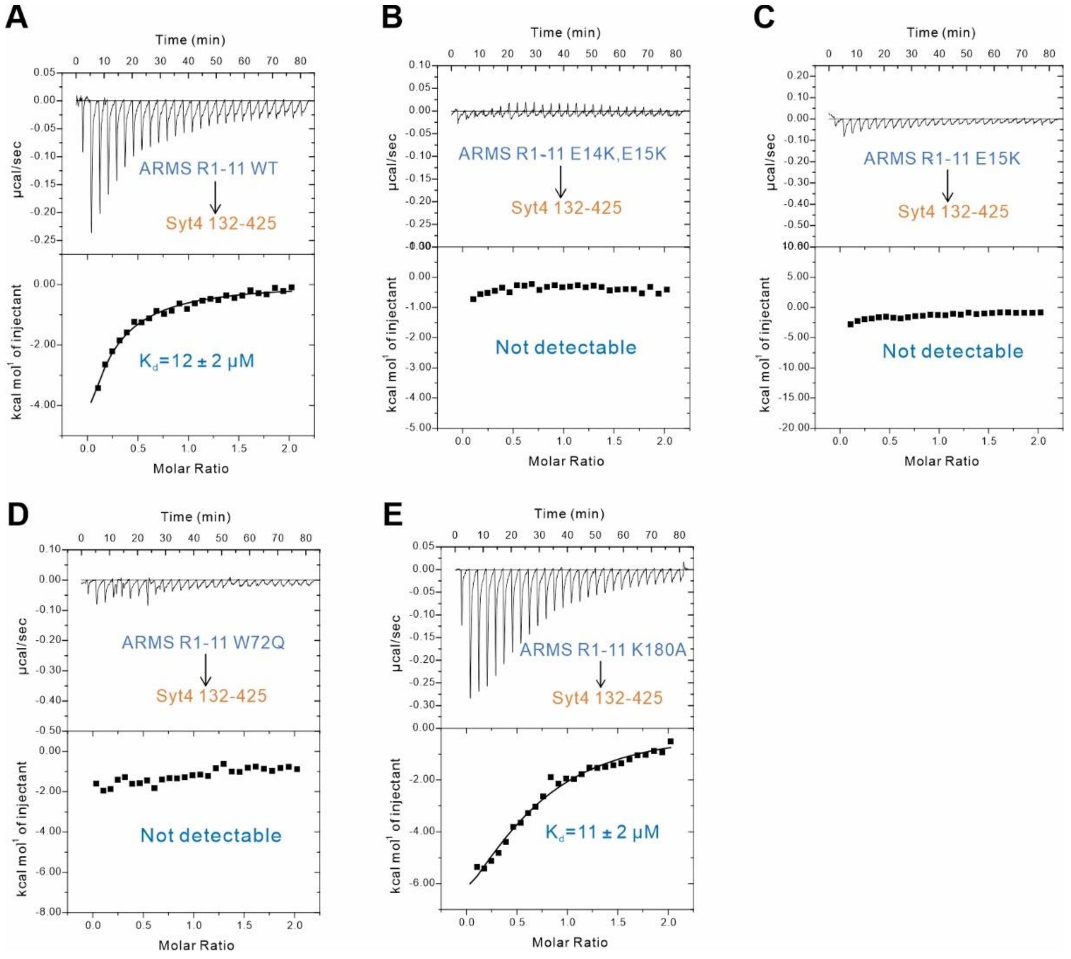

Figure S1. ITC results of the effect of mutations in ARMS on the binding with Syt4. Related to Figure 4C. **(A-E)** ITC-based measurements of the binding affinity of Syt4 132-425 with ARMS R1-11 WT **(A)**, E14K, E15K variant **(B)**, E15K variant **(C)**, W72Q variant **(D)**, and K180A variant **(E)**.

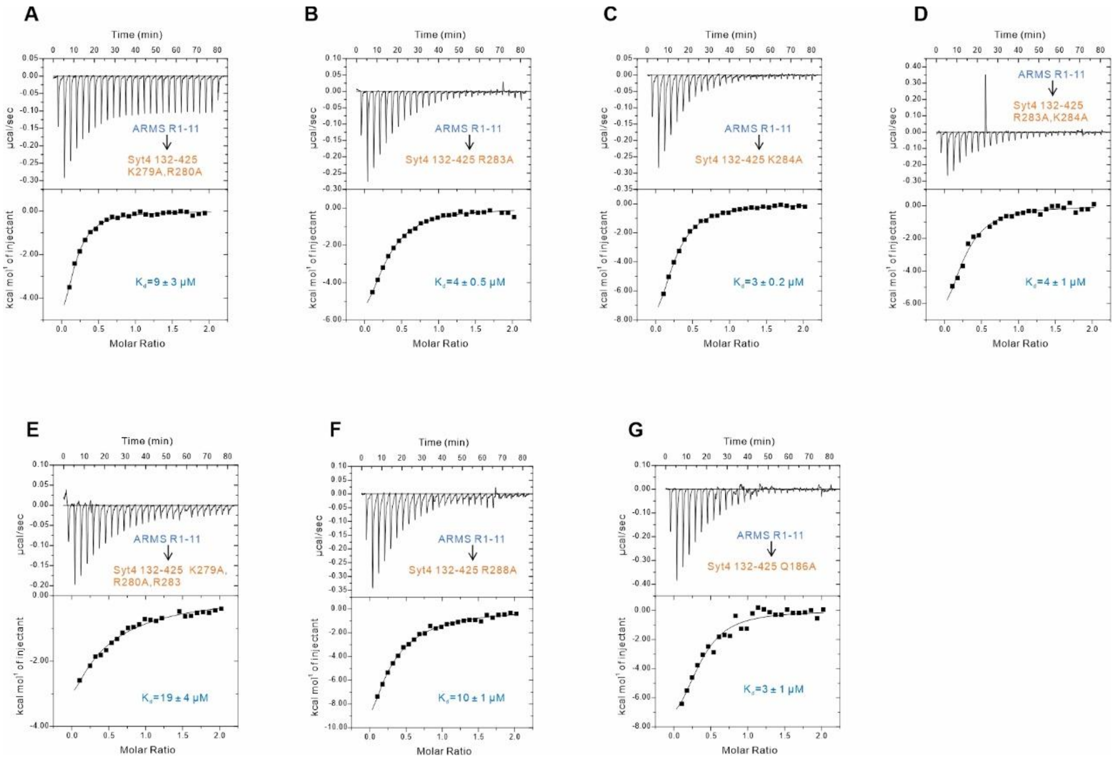

Figure S2. ITC results of the effect of mutations in Syt4 on the binding with ARMS. Related to Figure 4D.

(A-G) ITC-based measurements of the binding affinity of ARMS R1-11 with Syt4 132-425 K279A, R280A variant **(A)**, R283A variant **(B)**, K284A variant **(C)**, R283A, K284A variant **(D)**, K279A, R280A, R283A variant **(E)**, R288A variant **(F)**, and Q186A variant **(G)**.
